# Emerging pathological diagnostic strategies for solid pseudopapillary neoplasm of the pancreas: insights from omics and innovative techniques

**DOI:** 10.1002/2056-4538.70029

**Published:** 2025-05-01

**Authors:** Yuanhao Liu, Junya Peng, Yupei Zhao, Wenze Wang

**Affiliations:** ^1^ Department of Pathology Peking Union Medical College Hospital, Chinese Academy of Medical Science and Peking Union Medical College Beijing PR China; ^2^ Institute of Clinical Medicine Peking Union Medical College Hospital, Chinese Academy of Medical Science and Peking Union Medical College Beijing PR China; ^3^ State Key Laboratory of Complex, Severe, and Rare Diseases Peking Union Medical College Hospital, Chinese Academy of Medical Science and Peking Union Medical College Beijing PR China; ^4^ Department of General Surgery Peking Union Medical College Hospital, Chinese Academy of Medical Science and Peking Union Medical College Beijing PR China; ^5^ Department of Basic Medical Sciences School of Medicine, Tsinghua University Beijing PR China; ^6^ Peking University‐Tsinghua Center for Life Sciences Beijing PR China; ^7^ Molecular Pathology Research Center, Department of Pathology Peking Union Medical College Hospital, Chinese Academy of Medical Sciences and Peking Union Medical College Beijing PR China

**Keywords:** solid pseudopapillary neoplasm of the pancreas, precise diagnosis, omics

## Abstract

Solid pseudopapillary neoplasm (SPN) of the pancreas is a rare, low‐grade malignant tumor, representing 0.9–2.7% of all exocrine pancreatic tumors. SPN patients generally have a favorable prognosis with a 5‐year survival rate exceeding 95% following complete surgical resection. Accurate diagnosis is crucial to avoid unnecessary treatments. Currently, SPN diagnosis relies on imaging techniques such as CT and MRI, along with immunohistochemical analysis of biopsy and resection samples. The main challenge in diagnosis is the potential inability to accurately identify recurrent or metastatic SPN, as well as ‘malignant’ SPN, due to the lack of specific biomarkers. Advances in high‐throughput omics technologies, including genomics, transcriptomics, proteomics and metabolomics, have opened new avenues for identifying novel biomarkers for SPN. Additional, liquid biopsy techniques have enabled more comprehensive analysis of biosamples such as pancreatic cyst fluid, offering promising prospects for preoperative diagnosis. This review highlights recent research on SPN diagnosis, focusing on immunohistochemical markers, tissue sampling methods and the potential of omics approaches. It also discusses the challenges and opportunities in improving diagnostic accuracy, particularly for high‐grade and metastatic SPNs.

## Introduction

Solid pseudopapillary neoplasm (SPN) of the pancreas is a relatively rare, low‐grade malignant pancreatic tumor, accounting for 0.9–2.7% of all exocrine pancreatic tumors [1]. Unlike cystic pancreatic lesions such as intraductal papillary mucinous neoplasm (IPMN) and pancreatic mucinous cystadenoma (MCA), which often arise in older patients and carry a well‐defined risk of malignancy, SPNs predominantly occur in young females and exhibit distinct imaging features (e.g., heterogeneous solid‐cystic architecture with hemorrhage and calcification) [2]. Despite surgical resection being curative for most cases [3], the challenge lies in identifying the rare aggressive variants (malignant SPNs) through non‐invasive methods, which currently lack established biomarkers. Moreover, apart from conventional surgical resection, the systemic treatment options for this variant remain unclear. At the same time, there is a lack of efficient biomarkers to predict the recurrence and metastasis of SPN postoperatively, which further complicates patient management.

Advances in high‐throughput omics technologies, encompassing genomics, transcriptomics, proteomics, and metabolomics, have opened opportunities for identifying novel biomarkers for SPN. Furthermore, the development of liquid biopsy techniques has enabled more comprehensive analyses of additional biosamples from SPN patients, including pancreatic cyst fluid. These advancements present new possibilities for preoperative diagnosis of SPN.

**Figure 1:**
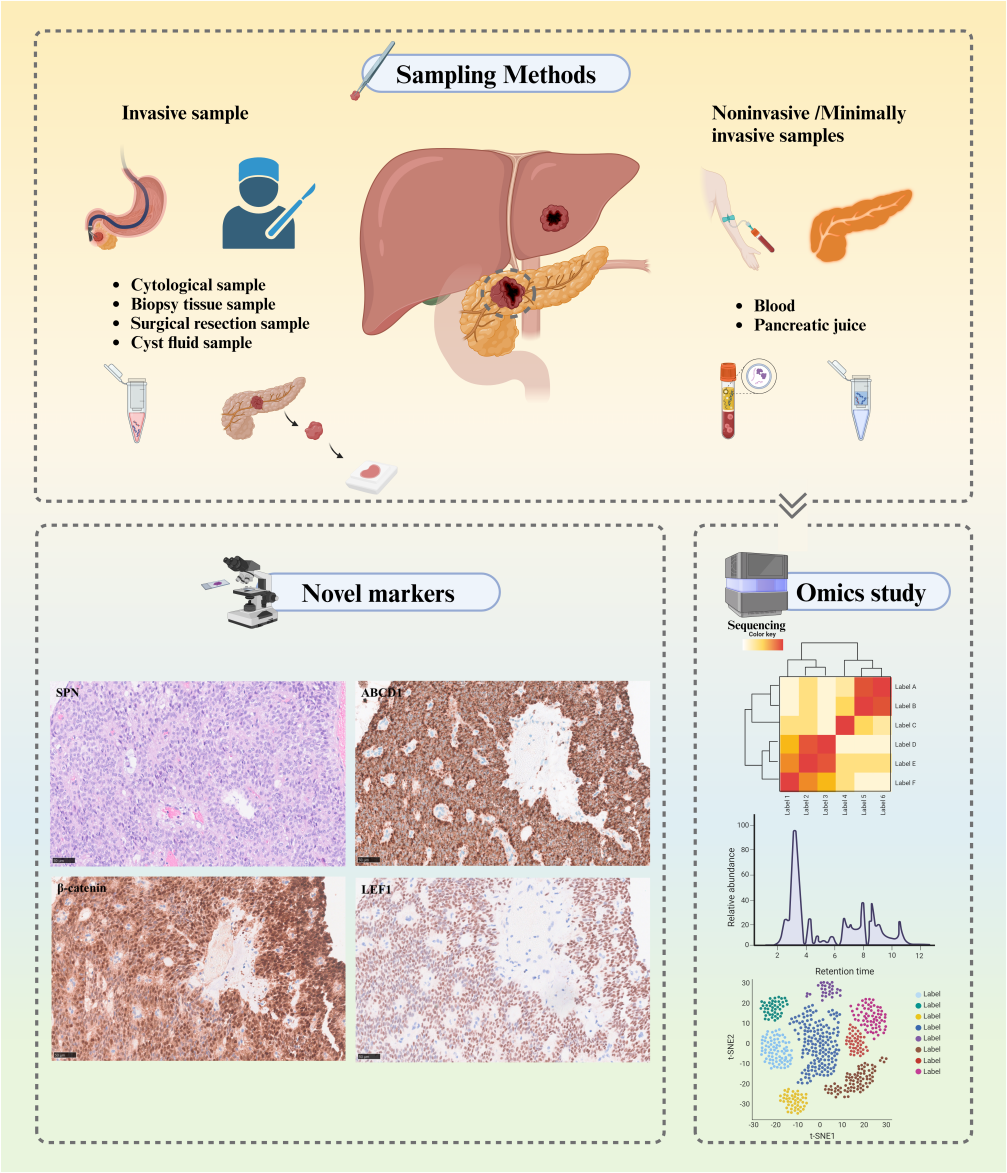
Overview of solid pseudopapillary tumor of the pancreas: sampling methods, novel markers, and omics studies. Created with BioRender.com

This review aims to summarize and analyze the latest research on the precision pathological diagnosis of SPN using various tissue samples obtained from invasive methods and minimally invasive methods. It will also highlight recent findings from various omics approaches related to SPN, discuss the associated opportunities and challenges, and suggest potential future research directions. The goal is to provide new perspectives and insights for the accurate diagnosis and comprehensive understanding of SPN (Figure 1).

## Biomarkers for the pathological diagnosis of SPN in clinical practice

In general, a diagnosis of SPN is largely suggested by imaging through the presence of a well‐demarcated heterogeneously hyperintense mass in the pancreas with MRI, accompanied by spontaneous high signal areas suggestive of internal bleeding [2]. Patients will undergo surgery without preoperative diagnosis. However, if these typical imaging features are not present, and due to the lack of diagnostic circulating biomarkers, preoperative diagnosis may require reliance on biopsy combined with immunohistochemical staining for confirmation. Pathologists rely on a variety of immunohistochemical markers in combination to diagnose SPN (Table 1).

**Table 1:** Diagnostic biomarkers for SPN

	Staining pattern	Positivity in SPN [reference(s)]	Positive expression in other pancreatic tumors
Positive markers			
β‐Catenin	Nuclear/Cytoplasmic	98.9–100% [4, 5]	ACC, PB
Cyclin D1	Nuclear	100% [4]	PanNET
Vimentin	Cytoplasmic	92% [6]	PanNET
PR	Nuclear	66.6–100% [7, 8]	PanNET
CD10	Cytoplasmic	87.5–100% [9, 10]	PanNET
CD99	Perinuclear	85.71–100% [8, 11]	–
CD56	Cytoplasmic	92.3–100% [7, 12]	PanNET
CD117	Cytoplasmic	50% [1]	IOPN
AR	Nuclear	81.3–100% [5, 7]	PanNET
Syn	Cytoplasmic	46.0–83.3% [7, 10]	PanNET
LEF1	Nuclear	93.4–94.2% [5, 13]	PB
ABCD1	Cytoplasmic	100% [14]	ACC, PB
P504s	Nuclear	96.2–100% [12, 15]	PanNET
CD138	Cytoplasmic	N/A	PanNET
CD200	Cytoplasmic/Membranous	100% [16]	PanNET
WIF‐1	Nuclear	96.7–100% [5, 9]	PanNET
TFE3	Nuclear	74.7% [5]	PanNET
AE1/AE3	Membranous	30–70% [1]	PDAC
Negative markers			
CK7	Membranous	N/A	PDAC
E‐cadherin	Membranous	* [17]	PDAC, ACC
CgA	Cytoplasmic	15.4% [15]	PanNET
Trypsin	Cytoplasmic	0% [18]	ACC
CEA	Membranous	N/A	PDAC
Bcl‐10	Cytoplasmic	0% [18]	ACC
CPA1	Cytoplasmic	5% [19]	ACC
REG1a	Cytoplasmic	0% [19]	ACC
INSM1	Nuclear	0–56% [20, 21]	PanNET
SSTR2	Membranous	N/A	PanNET
CEH	Cytoplasmic	0% [18]	ACC
Lipase	Cytoplasmic	0% [18]	ACC
Amylase	Cytoplasmic	0% [18]	ACC

–, negative or not evaluated on large series of other primary pancreatic neoplasms. N/A, not evaluated on large series of SPN.

ACC, acinar cell carcinoma; AR, androgen receptor; Bcl‐10, B‐cell lymphoma 10; CEA, carcinoembryonic antigen; CEH, carboxyl ester hydrolase; CgA, chromogranin A; CK7, cytokeratin 7; CPA1, carboxypeptidase A1; INSM1, insulinoma‐associated protein 1; IOPN, intraductal oncocytic papillary neoplasm; IPMN, intraductal papillary mucinous neoplasms; LEF1, lymphoid enhancer‐binding factor 1; P504s, α‐methylacyl CoA racemase; PanNET, pancreas neuroendocrine tumor; PB, pancreatoblastoma; PDAC, pancreatic ductal adenocarcinoma; PR, progesterone receptor; REG1a, lithostathine‐1‐alpha; SSTR2, somatostatin receptor 2; Syn, synaptophysin; TFE3, transcription factor E3; WIF‐1, WNT inhibitory factor 1.

* The immunohistochemical staining results depend on the binding site of the antibody used.

Over 90% of SPN cases exhibit a point mutation in exon 3 of the *CTNNB1* gene, leading to increased nuclear accumulation of β‐catenin and cyclin D1, along with a loss of E‐cadherin protein receptor from the cell membrane [22]. Consequently, nuclear positivity for β‐catenin has become a crucial immunohistochemical marker for diagnosing SPN, with nuclear positivity for cyclin D1 also observed [4]. The expression pattern of E‐cadherin in tumor cells varies depending on the antibodies used [17]. In addition, WIF‐1 is usually expressed positively in SPN [6, 9]. Additionally, the paranuclear dot‐like positivity of CD99 and nuclear positive expression of TFE3 serve as distinctive diagnostic clues for SPN [5, 11]. CD10, which has low specificity but high sensitivity, shows positive membrane expression in nearly all SPN cases [10]. More than 50% of SPN cases also exhibit positive expression for CD117, but it is also strongly expressed in intraductal oncocytic papillary neoplasm (IOPN) [23, 24]. In addition, CD138 and CD200 can also be positively expressed [16, 25]. Alpha‐methylacyl‐CoA racemase (AMACR, p504s) shows granular cytoplasmic expression [12, 15]. Both α1‐antitrypsin and α1‐antichymotrypsin demonstrate focal strong positive staining in small clusters of tumor cells within SPNs [26]. Some SPN cases may show features reminiscent of neuroendocrine differentiation, with 30–70% of cases displaying cytoplasmic positivity for CD56 and synaptophysin (Syn), but lacking chromogranin A (CgA), INSM1, and SSTR2 expression [1, 27]. Hormone expression in SPNs varies, with estrogen receptor (ER) expression being rare, while progesterone receptor (PR) and androgen receptor (AR) expression is notably higher [5, 7]. Moreover, SPN is negative for carboxypeptidase A1 (CPA1), regenerating islet‐derived 1α (REG1a) trypsin, Bcl‐10, and other markers derived from acinar cells [18, 19].

Despite the widespread use of immunohistochemical profiling for diagnosing SPN, accurate diagnosis of SPN remains challenging due to the morphological similarities and limited specificity of current markers. SPN can be easily mistaken for other pancreatic tumors, such as PanNET, pancreatoblastomas (PB), and acinar cell carcinomas (ACC) [28, 29, 30]. While SPN typically exhibits fibrovascular cores and pseudopapillary structures, these other tumors often present with solid or solid‐cystic structures. Their cells, usually round or oval with mild atypia, often form sheet‐like or nested patterns. Additionally, strong nuclear expression of β‐catenin is observed in these similar pancreatic tumors, further complicating the differential diagnosis [22]. Intra‐ and inter‐tumor heterogeneity can lead to variable intensity or even focal negativity for β‐catenin nuclear staining in SPN samples. This variability significantly increases the false‐negative rate in fine needle aspiration biopsies or cytological examinations, potentially resulting in misdiagnosis. Furthermore, the specificity of existing auxiliary markers for SPN diagnosis is limited. For example, while CD99 exhibits perinuclear dot‐like positivity in SPN samples with a sensitivity ranging from 85.71% to 100%, its specificity is only 48.21%, which is inadequate for clinical purposes [31, 32].

## Sampling methods and pathological evaluation of SPN

### Pathological evaluation of biopsy samples: advantages and disadvantages

Sampling of SPNs *via* needle biopsy primarily includes three types: fine‐needle aspiration (FNA), fine‐needle biopsy (FNB), and core needle biopsy (CNB). FNA typically uses needles of 25G or 22G, with diameters around 0.34 mm [33]. FNA samples are mainly suitable for cytopathology due to their small volume, which often limits the ability to assess the tumor's histomorphology. Although a limited number of immunohistochemical stains may be performed, the sample size usually precludes extensive molecular testing. FNB employs needles that are generally 22G or thicker, such as ProCore™, SharkCore™, and Acquire™, which have multi‐faceted tips or reverse bevels to capture more tissue than FNA [33, 34]. FNB samples are typically sufficient for both a complete series of immunohistochemical stains and further molecular analysis [35, 36]. CNB uses 18G needles, with a diameter of approximately 1.2 mm, providing the largest tissue samples among the three methods and thus enabling more comprehensive evaluations [37].

Beyond the amount of tissue obtained, factors such as tumor location, safety, and ease of operation influence the choice of biopsy method [38]. CNB is limited by the proximity of the lesion to major abdominal vessels, requiring a distance of greater than 1.5 cm between the lesion and vessels to minimize the risk of adverse events such as bleeding and pancreatitis [37]. Additionally, CNB demands a higher level of operator proficiency compared to FNA. Consequently, endoscopic ultrasound‐guided FNA (EUS‐FNA) and EUS FNB (EUS‐FNB) are more commonly employed in clinical practice.

While EUS‐FNB demonstrates a marginally higher diagnostic accuracy (87.5–100%) compared to EUS‐FNA (82.6–100%), this difference did not reach statistical significance (*p* = 0.16) [8, 39, 40, 41, 42]. However, current evidence supporting CNB in SPN diagnosis remains scarce, although an available report suggests that CNB exhibits a high diagnostic accuracy of 100% (2/2) [43]. Morphologically, typical SPN tumor cells from EUS‐FNA/FNB or CNB samples display a uniform appearance, often forming branching cell clusters with central capillaries [44]. In some cases, sieve‐like structures composed of small round cells with scant cytoplasm may be observed [45]. Rarely, multinucleated giant cells, clear cells, or foam cells may also be present [7]. Immunohistochemical staining of the positive and negative markers mentioned above is applied if the biopsy samples are sufficient. However, compared with CNB, the limited cellular yield from EUS‐FNA/FNB may necessitate additional operation for accurate diagnosis. For instance, a study of 52 patients who underwent EUS‐FNA found that 12 patients (21.15%) had adequate sampling for pathological evaluation [46]. Thus, there is a need to identify novel immunohistochemical markers with high specificity and sensitivity for use with limited tissue samples.

### Pathological evaluation of surgical resection samples

Surgical resection samples are the most commonly available specimens for diagnosing SPNs. These samples can be classified into three categories: primary lesions, aggressive tumors (referred to in this paper as ‘high‐grade SPNs’, which are SPNs with high‐grade carcinoma) and metastatic lesions. Typical non‐aggressive primary SPN resection samples consist of eosinophilic cells, clear cells, vacuolar cells, and spindle cells, forming distinct growth patterns including pseudopapillary, solid, microcystic, and trabecular [3]. In some cases, SPN variant tumor cells resemble those of hepatocellular carcinoma, presenting as well‐defined round nodules with focal foam‐like macrophage aggregates and clear globules [47]. Immunohistochemical markers, such as β‐catenin, CD10, and CD99 (positive markers), and Bcl‐10 and CgA (negative markers) are routinely employed to aid in the diagnosis.

Most SPNs are low‐grade malignant neoplasms with a favorable prognosis following complete surgical resection. However, high‐grade SPNs, which exhibit more aggressive characteristics, present a poor prognosis and may ultimately lead to patient mortality [13, 48, 49]. These high‐grade SPNs have increasingly garnered attention from clinical pathologists, not only for accurate diagnosis but also for identifying prognostic markers for malignant SPNs. Studies have highlighted several distinct features of high‐grade SPNs. Histologically, high‐grade SPNs share common pathologic characteristics with typical SPNs but also display additional malignant features, such as larger tumor size, coagulative necrosis, high‐grade nuclear atypia, lymphatic and/or venous invasion, and higher mitotic counts and Ki‐67 indices. Genetically, high‐grade SPNs often exhibit mutations in *CTNNB1* and alterations in tumor suppressor genes (*RB1*, *TP53*) as well as loss of heterozygosity (LOH) [48]. Currently, there are no established prognostic markers for predicting SPN malignancy.

Although most typical non‐aggressive SPNs have a generally good prognosis, local recurrence or metastasis can occur. Metastatic SPNs are rare and primarily affect the liver, abdominal cavity, and omentum. There is no definitive morphological feature that indicates the prognosis of SPNs, and metastatic lesions often still display uniform oval or round cells without malignant histological characteristics. The nuclear grade of the metastasis may be higher than that of primary tumors, and the pleomorphism may be more obvious [50]. Furthermore, some reports have noted that increased mitotic rates occur in metastatic tumor lesions [51]. Accurate diagnosis of the tumor origin in metastatic SPN lesions is essential for effective auxiliary diagnosis. Commonly used markers, along with additional CK20, CK7, Arg‐1, Glypican, and glutamine synthetase (GS) should be employed to elucidate tumor origin.

In conclusion, while surgical resection samples provide valuable insights into SPNs, the challenge lies in distinguishing high‐grade and metastatic SPNs from their less aggressive counterparts. This underscores the need for continued research to identify robust prognostic markers and improve diagnostic accuracy, particularly for high‐grade and metastatic SPNs.

### Major research advances based on cyst fluid samples

As a type of pancreatic cystic neoplasms (PCN), SPN differs from IPMN and pancreatic MCA, which have the potential to progress to malignant pancreatic ductal adenocarcinoma (PDAC) [52], in that it exhibits relatively less aggressive behavior. Therefore, accurate preoperative differentiation of SPN is crucial for effective management. Recent research has increasingly focused on molecular changes in cyst fluid samples obtained *via* EUS‐FNA, intraoperative collection, and endoscopic retrograde cholangiopancreatography (ERCP). Genetic panels targeting mutations in PCN‐related genes have demonstrated high diagnostic efficacy [53]. Although molecular studies on SPN cyst fluid remain limited, *CTNNB1* mutations (present in 100% of SPNs) [54] serve as a key distinguishing marker from IPMNs (GNAS/KRAS‐driven) and MCNs (KRAS‐driven). Although this research is still largely in its scientific stages, the integration of optimized and standardized cyst fluid sample collection technology, coupled with advanced metabolite and molecular testing, presents promising opportunities for enhancing preoperative diagnosis of SPN.

### Summary

While accurate diagnoses are often achieved, the limited tumor tissue available in biopsy samples necessitates the development of more sensitive and specific immunohistochemical markers. These markers should be applied to both cytological and biopsy samples to minimize the additional financial and physical burden on patients associated with repeated biopsies. Concurrently, surgically resected samples should be utilized to further explore aggressive and metastatic SPNs, with a focus on identifying markers of prognostic significance.

In addition to traditional samples, minimally invasive or non‐invasive methods, such as cystic fluid, blood, and pancreatic juice, are currently underutilized and insufficient for accurate preoperative SPN diagnosis. Incorporating multi‐omics sequencing and other molecular diagnostic techniques for these samples could significantly enhance preoperative diagnosis. For instance, detecting *CTNNB1* mutations in these non‐invasive samples might indicate the presence of SPN. Moreover, comparing typical SPN with high‐grade or invasive SPNs using non‐invasive samples could help identify new prognostic markers for the early detection of more malignant forms of SPN (Table 2).

**Table 2:** Sample types and status of clinicopathological diagnosis of SPN

Sample types	Acquisition methods	Pathological assessment		Utility	Limitations
		Typical pathomorphology	Special pathomorphology		
Invasive samples					
Cytological samples	EUS‐FNA	Branching clusters of tumor cells with central capillaries Morphologically uniform nucleus, occasionally with indented or grooved nuclear membranes. Bare nuclei may also be observed. Clean background or commonly hemorrhagic	Sieve‐like structures composed of small round cells with scant cytoplasm cercariform cells Multinucleated giant cells, clear cells, foam cells can also be observed.	Assessment of tumor malignancy preoperatively Preoperative diagnosis of SPN when sample yield is sufficient for IHC analysis	Increased operation times caused by insufficient sample yield Biopsy‐related complications
Biopsy tissue samples	EUS‐FNB; CNB under percutaneous ultrasound or CT guidance	Single and loosely cohesive tumor cells, arranged in a branching or papillary pattern. Round to oval nuclei, with dispersed or granular chromatin, and inconspicuous nucleoli. Nuclear grooves are commonly observed.	**–**	Preoperative diagnosis of SPN	Insufficient sample yield in some cases Limited assessment of tumor heterogeneity Biopsy‐related complications
Surgical resection samples	Surgical operation	Displaying four types of cells, including eosinophilic cells, clear cells, vacuolated cells, and spindle cells, with predominant growth patterns including pseudopapillary, solid, microcystic, and trabecular architectures. Round to oval nucleus, with dispersed or granular chromatin, and inconspicuous nucleoli. Nuclear grooves are commonly observed.	Special hepatocellular variant of SPN: histological resemblance to hepatocellular carcinoma, presenting as well‐defined round nodules with focal foam‐like macrophage aggregates and clear globules. Increased Ki‐67 index in metastatic SPN samples High‐grade SPN displaying characteristic of malignancy, tumoral necrosis, marked nuclear atypia, lymphatic and/or venous invasion, high mitotic count, and a high Ki‐67 index.	Typical strategy for SPN pathological diagnosis	Longer postoperative recovery time due to invasive procedures Postoperative complications Lack of accurate prognostic biomarkers for invasive SPN
Cyst fluid samples	EUS‐FNB; intraoperative collection; ERCP	(Laboratory only) *CTNNB1* gene alteration in molecular testing based on SPN cyst liquid samples	(Laboratory only) Presence of several gene alterations in other pancreatic cystic neoplasms: *MAPK*, *GNAS*, *VHL*, *TP53* or *TERT*, *SMAD4*, *PTEN*, and/or *RNF43*	Potential use in preoperative diagnosis of SPN	Laboratory only Lack of biomarkers for invasive SPN

## Opportunities and challenges of omics studies in SPN diagnosis

Recent advances in omics research have ushered in a new era of clinical problem‐solving through innovative technologies. Traditional omics approaches, including genomics, transcriptomics, proteomics, and metabolomics, along with emerging techniques such as epigenomics, single‐cell omics, and spatial omics, offer comprehensive, multi‐layered analyses. These methods provide insights into dynamic biological changes, from genetic alterations to protein levels, during disease progression. They not only elucidate the origins, genetic changes, metastasis, and prognosis of tumors but also facilitate the discovery of novel biomarkers to enhance clinical diagnosis. High‐throughput analyses of large‐scale data sets are critical for identifying disease‐related biomarkers, thereby advancing diagnostic capabilities.

### Molecular features of SPN based on genomic and epigenomic studies

The molecular features of SPN of the pancreas differ significantly from those of pancreatic ductal adenocarcinoma. Unlike the complex genetic landscape of PDAC, SPN is characterized by a relatively simpler mutation profile, with the highest mutation frequencies observed in the *CTNNB1*, *ATRNL1*, and *MUC16* genes [55]. SPN is primarily driven by genetic events in the Wnt signaling pathway, with 98.4% of cases exhibiting exon 3 mutations in the *CTNNB1* gene [55]. This mutation prevents the phosphorylation of β‐catenin observed in SPN but also leads to the upregulation of Wnt pathway target genes, such as *Cyclin D1*, *LEF1*, *AXIN2*, and *RNF43* [56]. These genetic alterations, in conjunction with other variations such as those in *USP9X, EP400*, *HTT*, *MED12*, and *PKD1*, may contribute to the pathogenesis of SPN [57].

Typical non‐aggressive SPNs exhibit a low proliferation rate, which is likely associated with the overexpression of cyclin‐dependent kinase inhibitors p21 and p27 [58]. However, the factors influencing this overexpression remain to be fully elucidated. Additionally, molecular changes, including single nucleotide polymorphisms (SNPs) and insertion–deletion mutations, have been widely detected in SPN [57]. In some cases, a frameshift deletion in the *APC* gene [c.3964delG p. (Glu1322Lysfs*93)] has been reported [59].

With advancements in sequencing technologies and a deeper understanding of SPN, recent research has focused on the molecular mechanisms underlying high‐grade and metastatic SPN. In high‐grade SPN cases, mutations beyond *CTNNB1* have been identified, including nonsense and frameshift mutations in *RB1*, LOH in the *RB1* gene, and a *TP53* p.Y107D mutation [48]. In metastatic SPNs, inconsistencies in *CTNNB1* mutation types between primary tumors and their matched metastases have been observed, suggesting clonal heterogeneity in the malignant progression of SPN [55, 57]. Moreover, alterations in genes such as *BAP1* and *KDM6A* have been proposed as potential drivers of SPN metastasis [29].

In addition to traditional genomic studies, preliminary research on SPN epigenomics has emerged in recent years. A 2022 study by Benhamida and colleagues analyzed genome‐wide DNA methylation levels in various pancreatic tumors, including SPN. The study found significant differential methylation of CpG probes in PDACs (35,968) and SPNs (34,042) compared to normal pancreatic tissue, indicating that the origin cells of SPN differ from the well‐known acinar, ductal, and neuroendocrine lineages [60]. These findings provide new insights into the unique molecular characteristics of SPN and its potential clinical use.

### Molecular features of SPN based on transcriptomic and proteomic studies

Transcriptomic studies conducted on SPN have demonstrated high expression of genes in the Wnt signaling pathway, such as ligands (*WNT5A, WNT2B, RSPO4*), receptor components (*LRG6, FZD10*), and other crucial genes for signal transduction like *APC*. Additionally, genes associated with the Notch pathway, including target genes (*HEY1, HEY2*), the Notch ligand gene (*JAG1*), and the receptor gene *NOTCH2*, are significantly upregulated in SPN [56, 61]. By comparing the differential gene expression in SPN with that in PDAC, PanNET, and non‐neoplastic pancreatic tissues, researchers identified the activation of the Hedgehog and androgen receptor signaling pathways, as well as genes involved in epithelial‐mesenchymal transition (EMT). Furthermore, 30 microRNAs were found to be specifically downregulated in SPN [9]. All these findings highlight potential key signaling pathways and genes, suggest a follow‐up functional validation, and translation into practical clinical applications.

To date, two key studies have been conducted in‐depth proteomic analyses of SPN, covering a total of 12 normal‐tumor tissue pairs and generating valuable public datasets for further research. The first study, by Zhu *et al*. in 2014, employed iTRAQ combined with liquid chromatography–tandem mass spectrometry (LC–MS/MS) to analyze five normal‐tumor tissue pairs from five different individuals. This study represents the first comprehensive analysis of the SPN proteome, producing a high‐confidence list of differentially expressed proteins. The findings highlight the potential association of the endoplasmic reticulum protein processing pathway with SPN pathogenesis [62]. The second study, conducted by Park *et al*., utilized high‐resolution mass spectrometry to analyze seven SPN tumor tissues compared to seven non‐neoplastic tissues. This study not only provided a valuable proteomic resource but also identified significant molecular variations in SPNs. These include upregulated proteins involved in the Wnt signaling pathway, such as DKK4, β‐catenin, and β‐catenin‐binding proteins FUS and NONO, as well as key enzymes in the glycolysis pathway, including PKM2, ENO2, and HK1 [63]. These molecular changes offer insights into the downstream molecular alterations in SPN.

### Molecular features of SPN based on scRNA‐seq profiling

Recently, single‐cell RNA sequencing (scRNA‐seq) has emerged as a powerful tool for characterizing cellular heterogeneity. In 2023, Meng and colleagues utilized scRNA‐seq to analyze four primary and one recurrent SPN samples, constructing the first comprehensive cell transcriptomic atlas of SPN. Ten major cell types were identified in primary SPN, including SPN tumor cells, dendritic cells, endothelial cells, fibroblasts, macrophages, monocytes, mast cells, B cells, T cells, and NK cells. Within SPN tumor cells, four heterogeneous meta‐programs were identified, which are related to stem cell proliferation, cell adhesion, granule lumen formation, and stress response. The study also provided insights into the pathogenesis of SPN, suggesting that SPN tumor cells may originate from pancreatic endocrine progenitor cells. A comparison between primary and recurrent lesions revealed upregulation of MYC‐related pathways and epithelial‐mesenchymal transition (EMT) pathways in recurrent SPNs. Furthermore, the study identified *NOV* and *DCN* as markers for primary SPNs and *S100A100* and *MGP* as markers for recurrent SPNs [64]. This discovery lays the foundation for the identification of novel diagnostic and prognostic biomarkers for SPN.

### Summary

Current omics research on SPN primarily focuses on bulk genomics, transcriptomics, and proteomics, but is limited by the lack of extensive datasets with large sample sizes. In‐depth studies of single‐cell omics are also scarce, particularly in adult cases. Furthermore, emerging omics fields such as metabolomics and microbiome analysis remain underexplored in SPN research. The limited availability of multi‐omics data for SPN is partly due to its low incidence. Additionally, the cystic or solid‐cystic nature of SPN, along with the presence of fibrovascular cores, necrosis, and calcification, complicates the purification of fresh tumor tissue. Future research should aim to address these gaps and broaden the application of diverse omics technologies.

As research on SPN progresses, identifying aggressive forms of SPN has become a key focus. Current studies primarily explore the relationships between clinical characteristics and patient outcomes, such as age, gender, tumor size, metastatic status (including lymph node involvement), positive tumor margins, and Ki‐67 index [13, 55, 65, 66, 67, 68, 69, 70, 71, 72]. However, specific thresholds for these factors vary across studies, particularly for tumor size and Ki‐67 index, likely due to differences in cohort sizes among cohort studies. For SPNs with high malignant potential, specific biomarkers associated with malignancy remain unidentified. Thus, a comprehensive understanding of SPN development, progression, recurrence, and metastasis is required through the integration of multiple omics approaches (Table 3).

**Table 3:** Main omics studies of SPN

	Year	Researchers	Cohort size	Specific techniques	Study conclusion
Genomics	2008	Rund *et al*. [73]	12 SPNs (10 adults, 2 children)	Array comparative genomic hybridization; Fluorescence *in situ* hybridization	Genetic alterations are common in SPN, especially in cases with aggressive features; Most common alterations were gains at 13q, 17q, 1q, and 8q.
	2017	Guo *et al*. [57]	9 SPN and 9 non‐neoplastic pancreatic tissues	NGS (WES)	54 SNPs and 41 indels of prominent variations were detected; A higher count of SNPs was particularly detected in patients with older age, larger tumor, and metastatic disease; *CTNNB1* mutations occur in all patients, which might collaborate with other events such as variations of *USP9X*, *EP400*, *HTT*, *MED12*, and *PKD1* to regulate tumorigenesis.
	2023	Liu *et al*. [55]	11 SPNs (7 Primary Tumors, 5 non‐neoplastic pancreatic tissues and 4 Paired Distant Metastases)	NGS(WES)	The three most frequently mutated genes were *CTNNB1*, *ATRNL1* and *MUC16*; The difference in gene alteration of *CTNNB1* between primary and matched metastatic samples suggests potential clonal heterogeneity during malignant progression of SPNs.
	2024	Honda *et al*. [48]	19 SPNs (4 HG‐SPNs and 15 non‐aggressive SPNs)	NGS (target DNA sequence); Sanger sequencing	All HG‐SPNs have *CTNNB1* mutations; Two HG‐SPN cases harbor *RB1* mutations with associated loss of heterozygosity (LOH), and immunohistochemical analysis revealed a complete loss of RB1 and diffuse overexpression of p16; Two HG‐SPN Cases also have *TP53* Mutations and/or p53 overexpression.
Epigenomics	2022	Benhamida *et al*. [60]	99 cases(12 SPNs, 9 NPTs and other Pancreatic Tumors)	NGS(methylation profiling)	Most SPNs are localized in distinct cellular clusters compared to PanNET, PDACs, and normal pancreatic tissues, while acinar‐derived tumors (ACCs and PBs) are found in overlapping regions between NPTs and PDAC clusters; PDAC (35,968) and SPN (34,042) exhibit the highest number of DMPs compared to NPTs.
Transcriptomics	2014	Park *et al*. [9]	31 cases (14 SPNs, 6 PDACs, 6 PanNETs and 5 non‐neoplastic pancreatic tissues)	NGS (RNA‐seq and small RNA‐seq)	The Wnt/β‐catenin, hedgehog, and androgen receptor signaling pathways, as well as genes involved in EMT, are activated in SPNs; 17 microRNAs, especially the miR‐200 family and miR‐192/215, closely associated with the upregulated genes associated with the three pathways activated in SPN and EMT.
Proteomics	2014	Zhu *et al*. [62]	5 SPNs and 5 non‐neoplastic pancreatic tissues	iTRAQ and LC–MS/MS	The top five up‐regulated proteins are hepatocyte growth factor‐like protein alpha chain, gelsolin, hemoglobin alpha‐2, doublecortin domain‐containing protein5, and hemoglobin beta chain. The top 5 down‐regulated proteins are a‐kinase anchor protein 11, zinc finger protein 714, hydrocephalus‐inducing protein homolog, cytochrome oxidase subunit2, and trypsin‐1; The top three pathways were the ribosome, protein processing in the endoplasmic reticulum, and the complement and coagulation cascades; Almost all subunits of the 60S and 40S ribosomal proteins are reduced in the SPN specimens.
	2015	Park *et al*. [63]	7 SPNs and 7 non‐neoplastic pancreatic tissues	iTRAQ, TMT, nano‐LC–MS/MS	A total of 329 differentially expressed proteins were found (150 up‐regulated and 179 down‐regulated). Among them, 58.1% of the proteins had the same expression tendencies in SPN according to the mRNA data; Increased levels of β‐catenin were observed, along with a significant up‐regulation of Wnt/β‐catenin signaling pathway proteins (DKK4) and β‐catenin‐binding proteins (FN1, SELENBP1, DDX5, YWHAZ, NONO, and FUS); Molecules involved in glycolysis, including *PKM2*, *ENO2*, and *HK1*, were overexpressed in accordance to their mRNA levels.
Single‐cell transcriptomics	2023	Meng *et al*. [64]	4 SPNs and 2 fetal pancreas specimens	Drop‐seq	The tumorigenesis of SPN may be closely related to oxygen metabolism, MYC targets, and DNA repair; SPN tumor cells may originate from pancreatic endocrine progenitor cells; *NOV*, *DCN* were nominated as primary and *S100A10*, *MGP* as recurrent SPN marker genes, respectively.

ACC, acinar cell carcinoma; DCN, decorin proteoglycan; DDX5, DEAD‐box helicase 5; DKK4, Dickkopf‐related protein 4; DMPs, differential methylation points; EMT, epithelial‐mesenchymal transition; ENO2, enolase 2; FN1, fibronectin 1; FUS, FUS RNA binding protein; HG‐SPN, high‐grade SPN; HK1, hexokinase 1; Indel, insertions and deletions; iTRAQ, isobaric tags for relative and absolute quantitation; LC–MS/MS, liquid chromatography–mass spectrometry; MYC, myelocytomatosis viral oncogene homolog; nano‐LC–MS/MS, nanoscale liquid chromatography coupled to tandem mass spectrometry; NONO, non‐POU domain‐containing octamer‐binding protein; NOV, nephroblastoma over‐expressed gene; NPT, normal pancreatic tissues; PanNET, pancreas neuroendocrine tumor; PB, pancreatoblastoma; PDAC, pancreatic ductal adenocarcinoma; PKM2, pyruvate kinase m2; scRNA‐seq, single cell RNA sequencing; SELENBP1, selenium binding protein 1; SNP, single nucleotide polymorphisms; TMT, tandem mass tag; WES, whole‐exome sequencing; YWHAZ, tyrosine 3‐monooxygenase/tryptophan 5‐monooxygenase activation protein zeta.

## Potential novel diagnostic biomarkers for clinical application in SPN

### LEF1

Missense mutations within exon 3 of the *CTNNB1* gene, as observed in SPN, inhibit phosphorylation, leading to cytoplasmic and subsequent nuclear accumulation of *CTNNB1*. Upon nuclear translocation, mutant *CTNNB1* interacts with the DNA‐bound lymphoid enhancer‐binding factor 1/T‐cell factor (LEF1/TCF) transcriptional complex [4]. In association with *CTNNB1*, LEF1 transactivates various Wnt‐responsive genes, including those that regulate the cell cycle and survival. Additionally, LEF1 plays a critical role in the epithelial‐mesenchymal transition (EMT) process by activating the transcription of N‐cadherin, Vimentin, and Snail molecules [74].

Recent studies have demonstrated strong nuclear expression of LEF1 in SPN. Although LEF1 shares a similar immunohistochemical staining pattern with the classical SPN diagnostic marker β‐catenin, research by Chen *et al*. found that LEF1 exhibits higher sensitivity (94.2%) and specificity (93.8%) in SPN compared to β‐catenin (sensitivity: 98.4%; specificity: 52.5%) [13]. This characteristic is also applicable in cytological samples [75]. The combination of LEF1 with INSM1 and PAX8 offers a new diagnostic approach for differentiating SPN from PanNET [20, 76].

However, due to LEF1's critical role in the Wnt pathway, it shares a diagnostic limitation similar to that of β‐catenin, particularly in PBs [77], where it also shows strong nuclear expression, complicating the accurate diagnosis of SPN. Additionally, as a diagnostic marker judged by nuclear positivity, deep cytoplasmic staining may cause misinterpretation of immunohistochemical results.

Current research has primarily focused on the differential diagnosis of LEF1 between SPNs and PanNETs, but larger cohort studies, potentially across multiple centers, are needed to verify LEF1 expression in other pancreatic tumors with morphological similarities to SPNs. Furthermore, it is worth investigating whether the level of LEF1 expression correlates with patient prognosis, and whether methylation of related gene promoters could serve as an early screening approach for SPN and other pancreatic tumors [78, 79].

### ABCD1

Recently, Yingao and colleagues identified significant upregulation of the peroxisome pathway in SPN for the first time. The peroxisome membrane protein ABCD1 was found to exhibit strong diffuse cytoplasmic positivity in SPN, demonstrating excellent specificity and sensitivity for distinguishing SPN from other pancreatic tumors such as PanNET, PB, and ACC (with a cut‐off value set at 1+, AUC = 0.999, sensitivity = 99.1%, specificity = 100%) [14]. The cytoplasmic localization of ABCD1 staining complements the nuclear positivity of β‐catenin. Moreover, ABCD1 showed only moderate cytoplasmic positivity in one case of PB (33.3%), helping to resolve potential diagnostic challenges posed by β‐catenin in other pancreatic tumors with Wnt pathway alterations. As a clearly distinguishable novel diagnostic marker, incorporating ABCD1 into the current immunohistochemical panel for SPN offers critical support for accurate diagnosis.

### Summary

Currently, although the two novel diagnostic markers have shown good sensitivity and specificity, the primary diagnostic markers for surgical resection samples still focus on β‐catenin, while new markers like LEF1 and ABCD1 remain largely within the realm of scientific research. Notably, compared to LEF1, ABCD1 is primarily expressed in the cytoplasm, which can help address the limitations of β‐catenin in clinical diagnosis by providing clearer cytoplasmic positivity. This supports the use of β‐catenin in clinical pathological diagnosis. Future efforts should aim to expand the application of ABCD1 in biopsy and cytological samples. Multicenter, large‐cohort prospective studies are needed to further validate its diagnostic efficacy in biopsy samples. This marker could then be combined with other existing immunohistochemical markers to create a limited, yet highly specific and sensitive panel, achieving both diagnostic accuracy and cost‐effectiveness.

## Author contributions statement

YL and JP were responsible for determining the scope of the topic, planning the literature search strategy and writing the first draft of the review. YZ and WW supervised and guided the whole manuscript.
